# Low Glycaemic Index Dietary Interventions in Youth with Cystic Fibrosis: A Systematic Review and Discussion of the Clinical Implications

**DOI:** 10.3390/nu4040286

**Published:** 2012-04-18

**Authors:** Ben W. R. Balzer, Christie L. Graham, Maria E. Craig, Hiran Selvadurai, Kim C. Donaghue, Jennie C. Brand-Miller, Kate S. Steinbeck

**Affiliations:** 1 Department of Adolescent Medicine, The Children’s Hospital at Westmead, Westmead 2145, Australia; Email: bbal8821@uni.sydney.edu.au; 2 Discipline of Paediatrics and Child Health, University of Sydney, Camperdown 2006, Australia; Email: maria.craig@health.nsw.gov.au (M.E.C.); hiran.selvadurai@health.nsw.gov.au (H.S.); kim.donaghue@health.nsw.gov.au (K.C.D.); 3 Department of Nutrition and Dietetics, The Children’s Hospital at Westmead, Westmead 2145, Australia; Email: christie.graham@health.nsw.gov.au; 4 Institute of Endocrinology and Diabetes, The Children’s Hospital at Westmead, Westmead 2145, Australia; 5 School of Women’s and Children’s Health, University of New South Wales, Randwick 2031, Australia; 6 Department of Respiratory Medicine, The Children’s Hospital at Westmead, Westmead 2145, Australia; 7 School of Molecular Bioscience, University of Sydney, Camperdown 2006, Australia; Email: jennie.brandmiller@sydney.edu.au; 8 Boden Institute of Obesity, Nutrition and Exercise, University of Sydney, Camperdown 2006, Australia

**Keywords:** cystic fibrosis related diabetes, glycaemic index, dietary intervention, adolescent

## Abstract

A systematic review was conducted to assess what is known about the effect of low glycaemic index (GI) diets on glycaemic control, weight and quality of life in youth with cystic fibrosis (CF). Eligibility criteria were systematic reviews, randomised and non-randomised trials of low GI dietary interventions in CF. Outcomes examined were glycaemic control, quality of life, anthropometry and respiratory function. Reference lists were manually searched and experts in the field were consulted. Four studies met the eligibility criteria; two were excluded because they did not include data on any of the outcomes. The remaining two were studies that examined GI secondary to any other intervention: one used GI as a factor in enteral feeds and the other incorporated low GI dietary education into its treatment methodology. There is insufficient evidence to recommend use of low GI diets in CF. Since there is evidence to support use of low GI diets in type 1, type 2 and gestational diabetes, low GI diets should be tested as an intervention for CF. The potential risks and benefits of a low GI diet in CF are discussed.

## 1. Introduction

Less than 25% of people with cystic fibrosis-related diabetes mellitus (CFRD) survive beyond 30 years of age, compared with 60% of CF individuals without CFRD [1,2]. CFRD is associated with deteriorating lung function [3] and an increased risk of bacterial chest infections, in part due to airway hyperglycaemia [4]. In addition, glucose intolerance negatively affects protein catabolism, which is accompanied by a decrease in body mass index (BMI) [5] and is also associated with an increased risk of deterioration in pulmonary disease [6]. As well as increased mortality [7,8,9,10,11], CFRD is associated with an increased need for lung transplantation, an increased treatment burden and potential risk of diabetic microvascular complications due to poor glycaemic control [12]. 

The prevalence of CFRD increases with age; from 20% at 15 years to 70% by age 30 [7]. CF with impaired glucose tolerance (CF-IGT) affects 20% of 10-year olds and 82% of the CF population by age 30 [7]. Clinical deterioration in CF has been described for up to six years prior to diagnosis of CFRD and exposed by more effective CF treatment [9,13]. 

CFRD is a distinct form of diabetes and its pathophysiology involves a gradual deterioration in insulin secretion [7,9,10] and diminished insulin sensitivity [6,7,9,13], which is accompanied by inflammation. Factors that may potentiate the risk of CFRD include the ΔF508 mutation [9], corticosteroid use [8] and chronic infection [11]. CF-IGT generally presents with normal fasting plasma glucose but abnormal post-prandial glucose levels [14]. Progressive damage to pancreatic beta-cells with continuing exocrine pancreatic destruction, combined with insulin resistance of puberty [15,16,17,18,19], likely explains the increased prevalence in CF-IGT and CFRD in adolescence. 

Insulin is considered the optimal therapy for CFRD [20,21], however the role of insulin for treatment CF-IGT remains controversial [13]. Insulin therapy in CF-IGT increases the treatment burden for people with CF and it remains to be established whether early initiation of insulin reduces long term morbidity and mortality [22]. 

The use of a low glycaemic index (GI) diet has shown potential for improving glycaemic control in other forms of diabetes [23], and is now recommended as part of dietary management in both type 1 and type 2 diabetes [24,25] Low GI foods contain carbohydrates that are more slowly digested and absorbed than higher GI foods, producing lower blood glucose excursions after consumption and consequently reduced endogenous insulin production [26]. Low GI diets are associated with improved insulin sensitivity, glycated haemoglobin and day-long glycaemia [23]. Low GI diets improve quality of life in type 1 and type 2 diabetes [23] and may have similar benefits in CF-IGT and CFRD. However, implementation of a low GI diet must incorporate the primary goals of nutrition therapy in CF: To achieve optimal weight gain (and growth in children and adolescents). Our aim was to systematically review the evidence base for use of a low GI diet in CF-IGT and CFRD.

## 2. Methods

**Figure 1:**
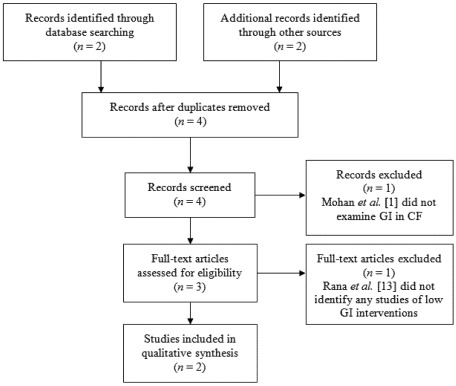
Study selection process.

A systematic search was conducted for low GI dietary interventions for CF or CF-IGT. We searched PubMed, Medline (via OvidSP), Embase, Web of Science and the Cochrane Database for Systematic Reviews for all years to January 2012. After devising search strategies and employing MeSH terms, searches were conducted for keywords and text (title or abstract) (Table 1). Search terms included: “cystic fibrosis”, “cystic fibrosis-related diabetes mellitus”, “impaired glucose tolerance”, “dietary intervention” and “glycaemic index”. Results were supplemented by manual searches of reference lists of other articles and journal indices. Additionally, experts in CF and endocrinology were consulted. Inclusion criteria were systematic reviews, randomised controlled or uncontrolled studies of low GI dietary interventions in patients with CF-IGT or CFRD. Data related to glycaemic control, quality of life, anthropometry or respiratory function after low GI dietary intervention was required for inclusion. Studies that did not report outcome data for a low GI intervention were excluded. BB and KS made the final decisions on inclusion/exclusion (Figure 1). 

**Table 1:** Search strategy for Medline (via OvidSP)

Search number	Search terms	Results
1	exp. Cystic Fibrosis/	26,002
2	Diabetes Mellitus/ or impaired glucose tolerance.mp.	88,034
3	exp. Glycemic index/ or glycaemic index.mp.	1661
4	1 AND 2	366
5	4 AND 3	2

## 3. Results

Four documents were recovered in the search. Two were recovered from the electronic search: Mohan *et al.* [1] and Skopnik *et al. * [27] and two from a manual search: Rana *et al. *[13] and Ntimbane *et al.* [28], a further two were excluded. Both articles recovered from the internet search were excluded: Mohan *et al.* [1] considered mechanisms of glucose tolerance was excluded because it did not examine GI in CF, and Rana *et al.* [13], a systematic review of CFRD management, discussed low GI diets as an option for improving glycaemic control [13], but it did not identify any studies of low GI dietary interventions. 

Skopnik *et al.* [27] examined the glycaemic response to enteral feeds in 19 adolescents (mean age 13 years) with CF, 11 previously shown to have normal oral glucose tolerance (CF-NGT) and 8 with abnormal oral glucose tolerance test (OGTT) results (CF-IGT or CFRD). Within a week of the initial 75 g oral glucose tolerance test (OGTT), the study participants were given an enteral feed of 1.75 g of carbohydrate per kg body mass (to a maximum of 75 g) and a carbohydrate tolerance test was performed using this enteral feed formula as the glucose challenge. The study compared the glycaemic load (GL), physiological response and glucose AUC to the enteral feed formula with the OGTT response. The enteral feed, which consisted of lower carbohydrate and higher fat and protein contents than the OGTT formula, had a lower GI (as measured by AUC) than the standard OGTT, presumably due to the lower glucose amount in the feed and the higher dietary fat and protein components within it [27]. Specific numerical data are not provided by the authors as data are AUC in graphic format. The authors report significant differences (*P* < 0.05) in blood glucose area under the curve between groups receiving the standard OGTT formula and the enteral feed over the course of the OGTT, with enteral feed leading to lower blood glucose levels regardless of glycaemic tolerance status. The study was non-randomised, and did not use a low GI dietary intervention.

Ntimbane *et al.* [28] was a two centre pilot intervention study of low GI dietary education *versus* no education, in children with CF, with primary outcomes related to tissue oxidative stress. Glycaemic status was only categorically evaluated by OGTT, but was not a primary end point of the study. Of the total study population (*n* = 31), 13 identified as having with CF-IGT of whom 1 dropped out of the study. Of the 12 remaining, nine were at one centre and received dietary education to avoid simple sugars and high GI foods. The three with IGT at the other centre did not receive this education. During 12 months follow up, the three who had not been educated all progressed to CFRD from CF-IGT. Of the nine who received education, two progressed to CFRD, six returned to CF-NGT and one remained CF-IGT. Lack of randomisation, the small sample size (*n* = 13) and no objective tool to measure dietary compliance are major limitations. To date, this study provides the only controlled evidence for a possible benefit of low GI diets in CF.

## 4. Discussion

The systematic review found no quality evidence for the use of low GI diets in CF and CFRD. Only two studies finally met the inclusion criteria, although neither examined a low GI intervention as a primary intervention in CF with IGT, the clinical scenario in which such diets are likely to have a use. Skopnik *et al.* [27] showed that a lower GI enteral feed formula provided a smaller AUC for glucose when compared to the standard OGTT. The lower GI enteral feed also led to improved glucose, C-peptide and insulin profiles under brief testing situation and allowed the authors to conclude that GI should be a factor for consideration in enteral feed selection [27]. Ntimbane *et al.* [28] provided some evidence in support of a low GI intervention for CF with IGT, although clearly these findings require confirmation in a more methodologically rigorous study. Thus our final conclusion must be that there is a dearth of evidence in support of, or against, the use of low GI diets and CF. None the less, the concept of a low GI intervention in CF deserves consideration for a number of clinically important reasons.

### 4.1. General Dietary Considerations in CF

Persons with CF require high-energy diets to maintain optimal nutritional status [29] as a result of their hyper-metabolic state. The increased energy requirements involve daily intakes of 120–150% of the recommended energy intake for the non-CF population [30] and the high energy density of fat is necessary. Generally, there are no restrictions on refined (high GI) sugars for those with CF, with carbohydrates forming close to half the daily energy intake. From clinical experience, the use of high glycaemic carbohydrates is common for snacks. The glycaemic excursion may vary, dependent for example on the concurrent fat intake. High fibre foods may be avoided because of bloating and the high satiety factor. Concern has been raised that dietary sugar may promote CF-IGT and CFRD [5,30], with high carbohydrate intake potentially exceeding insulin producing capabilities in CF. Although one study suggested the use of lower GI feeds to manage glucose intolerance [27], this has not been adequately trialled and is not suggested in guidelines for CF management. 

### 4.2. Low Glycaemic Index Diets as an Intervention in Non-CF Diabetes Mellitus

A low GI diet protects against development of type 2 diabetes [30,31,32,33] and conversely a high GI diet may contribute to the development of type 2 diabetes [29]. High GI diets are associated with increased insulin resistance [34] and interestingly a significant disruption of beta-cell architecture [35], findings which may be particularly relevant to CF. Average GI and GL (the product of GI × amount of dietary carbohydrate in grams) are significant independent predictors of type 2 diabetes risk in non-CF subjects [36]. 

Several clinical studies [29,30,31,32,37] and systematic reviews [23,26] have examined a low GI diet as an intervention in type 1 [30] and type 2 diabetes [32], as well as gestational diabetes mellitus [37]. In these studies, a low GI assisted in controlling glycaemia in both the short [31,32] and long [30] term. Most studies noted the ease in compliance [29,30,32,37], meaning it was a feasible intervention and one that can be implemented with a high degree of effectiveness and completion. Importantly, quality of life was not adversely impacted upon after a low GI intervention [23]. Low GI diets decreased post-prandial glycaemia and prevented hypoglycaemic events [30], and reduced exogenous insulin dose.

There are two studies of GI interventions in adolescents with the metabolic syndrome [31] or type 2 diabetes/IGT [32]. O’Sullivan *et al.* [31] demonstrated in an RCT that a decrease in GL of 20 units reduced by 2-fold the presence of the metabolic syndrome in adolescents. Palatability was discussed as a potential issue for compliance in this study, and the authors suggested that while a radical low GI intervention could be effective in adults, a slower transition to a lower GI and lower GL diet was needed in youth, with gradual substitution, though this did not appear to significantly alter their conclusions regarding the benefit of a low GI diet [31]. A crossover study in youth showed even one day of a low GI diet significantly decreased daytime glycaemia [32]. As with O’Sullivan *et al.* [31], abnormalities in glucose metabolism improved with a low GI dietary intervention, though the shorter duration and smaller sample size (*n* = 11) of this study is a limiting factor. Given, however, that these studies do not examine a CF population, one must exercise caution in extrapolating results and conclusions. As no current evidence base exists for low GI dietary interventions in CF, one must employ such results as potentially being applicable to CF until such a time that such an intervention in CF has been reported. 

### 4.3. The Potential for Weight Loss with a Low GI Diet

The Diet, Obesity and Genes (DiOGenes) Study, involved initial kilojoule restricted dieting, followed by two levels of protein and two levels of GI [38]. Both higher protein and lower GI diets were associated with protection from weight regain, with the best outcome for the high protein-low GI diet combination. Low GI dieters also had significantly better compliance and completion rate compared to their high GI counterparts [38]. These findings were mirrored in the extension of DiOGenes to children of the initial study subjects [39]. For CF patients, weight loss is to be avoided as it is associated with respiratory deterioration, so these findings must be of concern. However, in these studies, the focus was weight change and subjects were instructed to lose weight by overall calorie reduction [38]. The secondary outcome of improved glycaemic control [38,39] would be the intended outcome in the CF population but dietary prescription would need to be high energy and high dietary fat. 

### 4.4. Could a Low GI Dietary Intervention Delay Progression to CFRD?

A unique factor in CFRD pathogenesis, when compared to other forms of diabetes, is the origin of beta-cell loss. Inspissated mucus accumulation potentiates fibrotic damage by blocking the pancreatic exocrine duct system, leading to ischaemia and pancreatic endocrine tissue destruction. Common to CFRD and the other forms of diabetes mellitus, however, is hyperglycaemic toxicity to beta-cells [14,40]. Intervention studies have shown that hyperglycaemia-induced beta-cell apoptosis quickens the progression from IGT to type 2 diabetes [41,42]. While the endogenously-mediated destruction of the endocrine pancreatic tissue in CF is to-date non-modifiable, accumulation, the potential to modify hyperglycaemia-mediated beta-cell apoptosis should be considered as a means to slow the rate of decline in glycaemic tolerance.

The Skopnik *et al.* [27] and Ntimbane *et al.* [28] studies identified in this systematic review provide very preliminary evidence for the use of low GI diets in CF. Well designed, randomised controlled studies with primary outcomes related to GI are required. A benefit of early dietary intervention is the potential to delay insulin therapy, which introduces an additional treatment burden in CF. 

CF-IGT is a precursor of CFRD [5,7]. In CFRD, a low GI diet may reduce insulin requirements [32,37], particularly with acute infection or post-transplantation [21]. A low GI diet may also reduce the risk of postprandial hypoglycaemia, which can be problematic for individuals with CFRD. 

To measure the efficacy of any low GI diet in CF, multiple factors must be assessed, including anthropometry. While HbA1c and OGTT are likely to remain standard clinical measures of glycaemia, continuous subcutaneous glucose monitoring (CGM) [43] has particular relevance in CF, as glucose tolerance may vary over time and HbA1c measurement can be of limited value due to shortened lifespan of erythrocytes. CGM has been validated in youth with CF [44] and can detect clinically important hyperglycaemic events over a longer time frame than the two hour OGTT [45,46,47]. CGM is well tolerated [43,47] but has a higher cost compared to OGTT [43,47], which while limiting its clinical use, could be well justified under controlled study conditions. 

### 4.5. Concerns Related to the Use of Low GI Diets in CF

There are several concerns associated with a low GI diet, including weight loss, which has been partly addressed above (Section 4.3). An associated concern is that low GI may increase satiety [13,38] and this might reduce energy intake in CF. Therefore, a low GI diet in CF must be equivalent in energy to the current diet, and perhaps a “threshold” GI could be implemented, which enables a balance between improved glycaemic control and prevention of satiety.

Palatability, ease of consumption, cost and education burden all may play crucial roles in the adherence to any dietary intervention. Pertinent to adolescents especially is acceptance of the diet by peers and family, with peer acceptance likely to be predominant. In many cases, a low GI diet involves simply substituting food brands, and does not restrict the consumption of many dietary staples. In addition, the high fat content of many high sugar foods used in CF diets will render these foods low GI. Many low GI choices are available, including confectionery such as chocolate and peanut butter, which satisfy low GI requirements, are popular amongst youth and are energy dense because they are high in dietary fat. Given the importance of quality of life in the CF population, it too must be considered an important factor in such an intervention, no less because any reduction in quality of life may reduce the compliance rate in an adolescent population. As mentioned previously, low GI studies in a non-CF population have reported improvements to quality of life [23]. Additionally, if a low GI diet in adolescents with CF and CF-IGT can reduce the need for insulin, the associated reduction in treatment burden should increase or at least maintain quality of life. 

Enteral feeding has well established nutritional benefit in CF; however its high carbohydrate and high GL increases the risk of CFRD, with approximately half of enterally-fed adolescent subjects progressing to it in one study [22]. However, the authors note that causative relationships between enteral feeds and CFRD progression cannot be made from their results [22], so one must interpret this finding with caution. Any future interventional study using low GI foods would need to determine how best to integrate this therapy. 

A low GI diet may be more expensive if low GI foods are more expensive than high GI foods. There is a lack of evidence for cost effectiveness; therefore assessment of cost should be part of any low GI study in CF.

## 5. Conclusions

There is uncontrolled evidence from two studies demonstrating potential benefits of low GI diets, including improved glucose tolerance at one year follow up. Low GI diets are theoretically attractive and deserve scientific study as a possible dietary intervention in CF. The ease in switching to a low GI diet, as opposed to a proscriptive diet, means that the intervention should be relatively burden-free and therefore may meet with less resistance from individuals with CF, as almost all commonly eaten foods can still be enjoyed. An improvement in the glycaemic status of youth with CF, especially those with CF-IGT, may translate into improved quality of life and lower treatment burden in the longer term. 
